# Building an effective coverage cascade for antenatal care: linking of household survey and health facility assessment data in eight low- and middle-income countries

**DOI:** 10.7189/jogh.15.04048

**Published:** 2025-02-14

**Authors:** Safia S Jiwani, Saqib Rana, Elizabeth A Hazel, Abdoulaye Maïga, Emily B Wilson, Agbessi Amouzou

**Affiliations:** Department of International Health, Johns Hopkins Bloomberg School of Public Health, Baltimore, Maryland, USA

## Abstract

**Background:**

Substantial gaps exist between pregnant women’s contact with health facilities and the quality of care they receive (effective coverage) in low- and middle-income countries (LMICs). An effective coverage cascade is a useful analytical approach to uncover gaps due to poor facility service readiness and quality of care. We estimated readiness-adjusted antenatal care (ANC) coverage and built an effective coverage cascade in countries with available data.

**Methods:**

We used data from latest household and health facility surveys in eight countries accounting for 28 925 women and 8621 facilities. Service readiness was assessed based on the availability of core items needed to provide quality ANC. We linked the household surveys with health facility data by subnational region and facility type to estimate readiness-adjusted ANC coverage for at least one, four, and eight or more ANC contacts and ANC content. We built a four-step ANC effective coverage cascade and calculated loss of coverage in terms of ANC readiness coverage gaps and missed opportunities.

**Results:**

The majority of women sought ANC services in lower-level facilities, except in Bangladesh, Nepal and Senegal. While at least one antenatal care contact (ANC1+) service coverage was high, ranging from 89.2% (95% confidence interval (CI) = 87.2–90.9) in Haiti to 98.1% (95% CI = 97.5–98.6) in Malawi, readiness-adjusted ANC1+ coverage was lower, ranging from 64% (95% CI = 62.4–65.5) in Haiti to 76.2% (95% CI = 75.1–77.2) in Nepal. We obtained readiness gaps as high as 33.7 percentage points in Malawi and missed opportunities of 21 percentage points in Tanzania. Poor diagnostic capacity and insufficient trained human resources drove the low ANC facility readiness. We found large inequalities in readiness-adjusted ANC1+ by socioeconomic status favouring wealthier and urban resident women.

**Conclusions:**

The effective coverage cascade for ANC services helped uncover large readiness gaps, missed opportunities, and socioeconomic inequalities. Improvements in facilities’ diagnostic capacity and availability of trained human resources will enhance their ability to provide high quality health services and ensure health gains.

Service coverage is defined as the proportion of a population in need of a health service who received the service. While coverage indicators have been widely used to monitor progress in achieving health targets such as the Sustainable Development Goals, many fall short in incorporating the quality of services received, beyond tracking mere contact with health facilities [1]. In that endeavour, effective coverage measures have been proposed as actionable metrics incorporating aspects of service need, use, and quality, in order to better inform health gains and monitor universal health coverage [1,2]. Multiple definitions of effective coverage have been proposed, some more complex than others, but all trying to assess the proportion of individuals who received quality care to satisfy their specific service need [2].

A useful approach to operationalise the effective coverage concept is through the coverage cascade framework that illustrates the loss of effective service coverage experienced by a target population in need of a service. One such framework, proposed by Amouzou et al. [1], builds on the Tanahashi framework [3] and has been used for services of reproductive, maternal, newborn, child health and nutrition. This cascade is organised in a step-wise fashion following seven key steps:

1) the population in need of a service (the target population)

2) service contact, or the proportion of target population visiting the health service

3) likelihood of service, or the proportion that visits a facility that is ready for the service

4) crude coverage or the proportion that actually receives the health service

5) quality-adjusted coverage, or the proportion that receives health services according to quality standards

6) user-adjusted coverage reflecting user-adherence

7) outcome-adjusted coverage reflecting the health gain achieved.

Hence, this framework allows quantifying the losses to effective service coverage that occurs between each step of the cascade, including facility readiness gaps between steps two and three, and missed opportunities between steps three and four [1].

Antenatal care (ANC) is a critical intervention to prevent and treat pregnancy-related complications, as well as offer counselling on birth preparedness and ensure a positive pregnancy experience for both the mother and the baby [4]. According to the WHO, high quality ANC by a skilled health provider should include risk identification, prevention and management of pregnancy-related or concurrent diseases, as well as health education and health promotion. It is therefore an essential platform to detect women at increased risk of developing pregnancy complications. The most recent WHO guidelines also recommend attaining at least eight ANC contacts (ANC8+) during pregnancy, with the first contact occurring during the first trimester of gestation [4]. While most indicators tracking progress towards meeting maternal health targets focus on the number of ANC contacts achieved, less evidence exists on the content of care received, how complete the interventions are, and whether the facilities attended have the needed capacity, equipment, infrastructure and human resources to offer high quality care.

To fill this important gap, our aim was to estimate readiness-adjusted ANC coverage and build an effective coverage cascade for ANC in eight low-and middle-income countries (LMICs) with available data, based on the framework proposed by Amouzou et al. [1]. We also calculated loss of coverage by estimating the readiness coverage gap and missed opportunities, and we assessed socioeconomic inequalities in at least one antenatal care contact (ANC1+) coverage and readiness-adjusted coverage.

## METHODS

### Study design and data

We conducted a cross-sectional analysis of the coverage of ANC among women of reproductive age (15–49 years) with a birth in the previous two years, using household survey data from eight countries: Bangladesh, Haiti, Kenya, Malawi, Nepal, Niger, Senegal, and Tanzania. We used Demographic and Health Surveys (DHS) [5] in all countries except in Niger where we analysed the national survey on fertility and child health conducted in 2021 [6]. Demographic and health surveys were implemented between 2015–2022.These are nationally representative household surveys implemented in over 90 LMICs, providing data on health and population indicators. Relevant to our analysis, women of reproductive age with at least one recent pregnancy are interviewed on care seeking during their most recent pregnancy, including the number of ANC contacts received, the place of ANC, and specific interventions received during ANC. A total of 29 925 women of reproductive age with a recent birth were interviewed in the household surveys, ranging from 1005 in Senegal to 7280 in Kenya.

We used facility inventory data from health facility assessments from each of these countries (Service Provision Assessments [7], Service Availability and Readiness Assessments [8], and Harmonized Health Facility Assessments [9]) to estimate a readiness index for ANC services. The facility assessments were conducted between 2014–2021, and covered a total of 8621 health facilities, ranging from 285 in Senegal to 2433 facilities in Kenya. Facilities were either sampled or assessed through a national facility census.

The eight countries were selected based on data availability: we identified surveys that allowed linking the household survey with the health facility assessment with adequate temporality. We restricted the analysis to nationally representative health facility assessments conducted within 2–3 years of the household survey, in order for the mother’s report of ANC to reflect the care received in the facility within this time period. We assumed that facility readiness remains relatively constant during this time period [10,11]. Of all the countries with household surveys conducted between 2015–2022, only eight met the criteria of having a nationally representative health facility assessment conducted within 2–3 years of the household survey.

While each countries’ context was unique, they shared similarities in their free maternal health services policies and health care system characterised by a mix of public and private (for-profit and non-profit) facilities. Typically, the primary-level facilities (health centres or similar facilities) act as the first point of contact with women for ANC services, followed by hospitals at local/district-level, provincial and national levels; the latter include specialised and referral hospitals.

### Statistical analysis

#### ANC coverage and content: household survey

Three ANC coverage indicators were defined as the proportion of women of reproductive age (15–49 years) with a birth in the two years preceding the survey who reported receiving ANC1+, at least four ANC contacts (ANC4+) and ANC8+. In addition, to assess the number of ANC interventions received, we generated an ANC content score, reflecting the number of ANC content interventions reported to have been received by women, out of the following six interventions that should have been received during pregnancy as part of ANC: blood pressure measurement, blood test, urine test, counselling for pregnancy complications, intermittent prevention treatment for malaria during pregnancy (two + doses of Sulfadoxine Pyrimethamine), and iron and folic acid supplementation. The selection of these six interventions was entirely based on data availability and reflecting content of ANC based on programme implementation across the eight countries; it was not meant to cover the exhaustive list of interventions that women might receive during ANC in each country. When an intervention was not relevant/implemented or collected in a given country, we based the ANC content score on the total number of interventions collected in that country. The score was rescaled to 100 and expressed as a percentage for ease of interpretation and comparison across countries.

#### ANC readiness: health facility assessment

Using health facility surveys, we assessed facility readiness for ANC services by generating an ANC readiness score, reflecting the availability and functionality of 19 essential items across five domains needed for ANC service provision, informed by Sheffel et al.’s expert survey [12]: equipment and supplies, diagnostics, medicines, basic amenities, and human resources (Table S1 in the **Online Supplementary Document**). The score was computed as a simple additive measure, calculated as the arithmetic average of the proportion of items available out of the total number of items considered in each domain. The score ranged from 0–1 and was rescaled to a percentage between 0–100%.

#### Ecological linking: household survey with facility assessment data

We conducted an ecological linking analysis of household survey and health facility assessment data: for each country, a facility crosswalk was developed to match the ANC facility stratum (facility type and managing authority) reported by the woman from the household survey to the corresponding facility type and managing authority available in the health facility survey. For women who reported more than one ANC facility types, we recorded the highest level. We then linked a woman’s report of: a) ANC contact and b) ANC content from a given facility stratum (from the household survey) to the average facility ANC readiness score of that facility level in her geographic region of residence (from the facility assessment survey), thus generating readiness-adjusted ANC coverage and readiness-adjusted ANC content estimates. Women who did not receive any ANC contact during their last pregnancy, and those who attended ANC in informal facilities (home or other informal sector) were assigned a readiness score of zero; the proportion of such cases ranged from 2.6% of the sample in Malawi to 12.1% in Haiti.

#### Effective coverage cascade for ANC

From the above generated estimates, we built an effective coverage cascade for ANC, using the framework and definitions proposed by Amouzou and colleagues [1]. We adapted it to a four-step ANC cascade:

1) the first step is the target population in need of ANC services (all pregnant women)

2) the second step is the service contact, or coverage of ANC1+ services, generated from household survey data

3) the third step is the readiness-adjusted ANC1+ coverage, estimated by linking household survey with health facility assessment data

4) the last step is the readiness-adjusted ANC content, also issuing from the linking analyses.

From this cascade, we assessed the ‘lack of access or awareness’ as the absolute difference, in percentage points (pp), between the population in need of ANC services and ANC1+ coverage. The ‘ANC readiness gap’, defined as the difference between a woman visiting a facility and a woman visiting a facility that is ready for ANC services [1], was calculated as the absolute difference in pp, between ANC1+ coverage (the service contact) and the readiness-adjusted ANC1+ coverage. The ‘ANC missed opportunity’, defined as the difference between a woman’s visit of a health facility that is ready for the service, and the actual receipt of that service [1], was calculated as the absolute difference in pp between the readiness-adjusted ANC1+ coverage and the readiness-adjusted ANC content coverage. We did not use ANC4+ and ANC8+ coverage measures in the effective coverage cascade given we had one facility readiness estimate linked to a woman’s report of overall ANC, regardless of the number of ANC contacts she received. Additionally, ANC content interventions were not collected for specific ANC contacts, but rather during a woman’s pregnancy.

Lastly, we assessed socioeconomic inequalities in the ANC1+ coverage and readiness-adjusted coverage by women’s household wealth and their place of residence, collected in the DHS data.

## RESULTS

### Where did women seek ANC services?

The place of ANC varied by country. However, in most cases, a larger proportion of women received ANC services in low-level primary care facilities: in Niger, 88.3% of women reported receiving ANC from or public health centres (centres de santé integrés); in Tanzania and Malawi, 55.2 and 62.6% respectively received ANC from dispensaries, a type of primary care facility. In contrast, in settings such as Bangladesh, Nepal, and Senegal, a large proportion of women reported utilising private and public hospitals for ANC services: in Bangladesh and Senegal more than half of women reported receiving ANC from private hospitals (Figure S1 in the **Online Supplementary Document**).

### Antenatal care coverage and content

**Figure 1:**
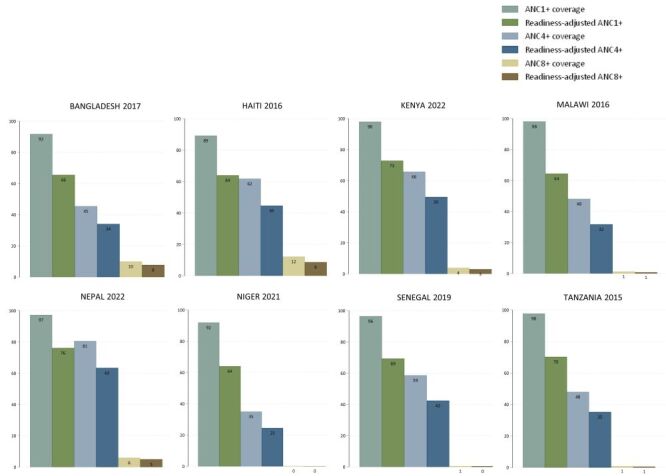
Coverage and readiness-adjusted coverage for ANC1+, ANC4+ and ANC8+, by country. ANC – antenatal care, ANC1+ – at least one antenatal care contact, ANC4+ – at least four antenatal care contacts, ANC8+ – at least eight antenatal care contacts.

Coverage of ANC1+ was near universal in all countries, ranging from to 89.2% in Haiti in 2016 (95% CI = 87.2–90.9) to 98.1% (95% CI = 97.5–98.6) in Malawi in 2016. Coverage for ANC4+ was lower, ranging from 35% in Niger in 2019 (95% CI = 32.5–37.5) to 80.6% in Nepal in 2022 (95% CI = 77.9–83.0). Coverage of ANC8+, the current WHO recommendation for a positive pregnancy experience, was very low at under 12% in all countries (**Table 1**, **Figure 1**). Content of care was generally high, with scores ranging from 67% (95% CI = 65.6–68.6) in Tanzania to 92% (95% CI = 90.9–93.1) in Nepal (**Table 1**). How ready were facilities to provide antenatal care services?

**Table 1:** Antenatal care coverage and readiness-adjusted coverage

	Service coverage				Readiness-adjusted service coverage				Lack of access	Readiness gap	Missed opportunities
**Country, year (n = sample of women)**	(a) ANC 1+ % (95% CI)*	(b) ANC 4+ % (95% CI)*	(c) ANC 8+ % (95% CI)*	(d) ANC content score* % (95% CI)*	(e) Readiness-adjusted ANC1+ % (95% CI)*	(f) Readiness-adjusted ANC4+ % (95% CI)*	(g) Readiness-adjusted ANC8+ % (95% CI)*	(h) Readiness-adjusted ANC content % (95% CI)*	**100–(a) (pp)**	**(a)–(e) (pp)**	**(e)–(h) (pp)**
Bangladesh, 2017 (n = 3411)	91.7 (90.4–92.9)	45.5 (42.9–48.0)	10.0 (8.7–11.3)	78.6 (77.2–80.1)	65.5 (64.1–67.0)	34.1 (32.0–36.1)	7.8 (6.8–8.8)	58.3 (56.9–59.6)	8.3	26.2	7.2
Haiti, 2016 (n = 2316)	89.2 (87.2–90.9)	61.8 (58.6–65.0)	12.2 (10.4–14.0)	87.1 (85.1–89.0)	64.0 (62.4–65.5)	44.6 (42.2–47.1)	8.6 (7.4–9.9)	63.1 (61.4–64.8)	10.8	25.2	0.9
Kenya, 2022 (n = 7280)	98.0 (97.6–98.3)	65.8 (64.0–67.5)	3.8 (3.2–4.6)	79.1 (78.8–79.4)	72.9 (72.3–73.4)	49.5 (48.0–51.0)	3.0 (2.4–3.5)	58.9 (58.4–59.4)	2.0	25.1	14
Malawi, 2016 (n = 6521)	98.1 (97.5–98.6)	48.2 (46.4–49.9)	1.2 (0.8–1.7)	72.3 (71.6–73.1)	64.4 (63.38–65.1)	31.7 (30.5–32.9)	0.7 (0.5–1.0)	47.8 (47.1–48.4)	1.9	33.7	16.6
Nepal, 2022 (n = 1928)	97.3 (96.1–98.1)	80.6 (77.9–83.0)	6.0 (4.5–7.8)	92.0 (90.9–93.1)	76.2 (75.1–77.2)	63.4 (61.3–65.5)	5.0 (3.5–6.3)	72.5 (71.3–73.7)	2.7	21.1	3.7
Niger, 2021 (n = 3339)	92.0 (89.5–93.9)	35.0 (32.5–37.5)	0.3 (0.1–0.5)	69.0 (67.0–71.0)	64.1 (62.5–65.6)	24.5 (22.7–26.3)	0.2 (0.0–0.3)	48.2 (46.7–49.7)	8.0	27.9	15.9
Senegal, 2019 (n = 1005)	96.5 (95.2–97.4)	58.6 (52.9–64.3)	0.5 (0.0–1.3)	87.3 (85.8–88.8)	69.3 (68.0–70.7)	42.4 (38.1–46.7)	0.4 (0.0–1.1)	62.8 (61.3–64.3)	3.5	27.2	6.5
Tanzania, 2015 (n = 4125)	97.8 (97.0–98.3)	48.0 (45.8–50.2)	0.8 (0.5–1.2)	67.1 (65.6–68.6)	70.3 (69.4–71.2)	35.3 (33.6–37.0)	0.6 (0.3–0.9)	49.2 (47.8–50.6)	2.2	27.5	21.1

ANC – antenatal care, CI – confidence interval, pp – percentage points

*ANC content was based on intervention relevance and data availability in each country as follows: Bangladesh: urine test, blood test, blood pressure, iron/folic acid supplements; Haiti: urine test, blood test, blood pressure, iron/folic acid supplements; Kenya: urine test, blood test, blood pressure, iron/folic acid supplements, Intermittent preventive treatment in pregnancy for malaria (IPTp); Malawi: urine test, blood test, blood pressure, iron/folic acid supplements, IPTp; Nepal: urine test, blood test, blood pressure, iron/folic acid supplements; Niger: urine test, blood test, blood pressure, iron/folic acid supplements, IPTp; Senegal: urine test, blood test, blood pressure, iron/folic acid supplements, IPTp, counselling on pregnancy complications; Tanzania: urine test, blood test, blood pressure, iron/folic acid supplements, IPTp.

### Facility readiness for ANC

Facility readiness for ANC varied across countries and by facility type. The mean ANC readiness score ranged from 58.7% (95% CI = 57.2–58.9) in Bangladesh, to 75.3% (95% CI = 73.9–76.7) in Senegal. Readiness scores by domains suggested that in the majority of countries, equipment items were most readily available, whereas diagnostics and human resources were lagging, with lowest domain-specific readiness scores pulling the overall score down (**Table 2**). Looking at the overall score by facility type (Figure S1 in the **Online Supplementary Document**), facility readiness was generally higher in upper-level facilities such as hospitals and clinics, and more variable in lower-level facilities (health centres, dispensaries, health posts/huts). Zooming in further of specific items needed for high quality ANC, Figure S3 in the **Online Supplementary Document** suggests that across countries, diagnostic testing for haemoglobin, syphilis, as well as urine dipsticks were lacking in the majority of countries; similarly, the proportion of staff trained in ANC in the last two years was low in all countries.

**Table 2:** Facility readiness for antenatal care services

		ANC readiness: overall and domain scores					
**Survey, year**	**Facilities, n**	**Overall score % (95% CI)**	**Equipment % (95% CI)**	**Diagnostics % (95% CI)**	**Medicines and commodities % (95% CI)**	**Basic amenities % (95% CI)**	**Human resources % (95% CI)**
Bangladesh, 2017	1495	58.7 (57.2–58.9)	74.5 (73.1–76.1)	17.5 (15.7–19.3)	63.7 (62.6–64.8)	79.2 (77.4–81.0)	25.0 (21.1–28.0)
Haiti, 2016	922	67.6 (66.7–68.5)	83.1 (82.0–84.3)	44.7 (42.7–46.7)	66.4 (64.5–68.3)	78.2 (76.7–79.7)	44.8 (42.5–47.1)
Kenya, 2019*	2433	70.2 (69.6–70.8)	85.0 (84.3–85.7)	34.5 (33.4–35.6)	86.8 (85.4–88.3)	89.0 (88.2–89.8)	N/A
Malawi, 2016	643	67.4 (66.4–68.3)	73.9 (72.2–75.6)	37.4 (35.7–39.0)	89.4 (87.9–90.9)	89.9 (88.5–91.3)	37.5 (35.1–39.9)
Nepal, 2021	1494	67.4 (66.5–68.2)	89.7 (88.7–90.7)	30.5 (28.1–33.0)	74.7 (73.4–76.1)	89.0 (87.5–90.4)	8.3 (7.2–9.3)
Niger, 2019	318	69.6 (68.1–71.0)	93.4 (92.0–94.7)	34.3 (31.0–37.5)	78.3 (75.1–84.5)	83.6 (80.9–86.2)	38.5 (33.0–44.0)
Senegal, 2019	285	75.3 (73.9–76.7)	76.2 (73.5–78.9)	80.2 (77.3–83.1)	86.3 (80.1–92.5)	71.3 (68.6–74.1)	23.6 (17.2–30.1)
Tanzania, 2014	1031	68.4 (67.3–69.6)	78.5 (77.0–80.2)	46.9 (44.5–49.2)	92.0 (90.4–93.6)	68.6 (66.3–70.9)	34.2 (31.8–36.7)

ANC – antenatal care, CI – confidence interval

*Overall readiness score in Kenya excludes human resources domain due to data unavailability.

### Readiness-adjusted coverage of ANC

The readiness-adjusted ANC1+ coverage ranged from 64.0% in Haiti (95% CI = 62.4–65.5) to 76.2% in Nepal (95% CI = 75.1–77.2), whereas that of ANC4+ ranged from 24.5% in Niger (95% CI = 22.7–26.3) to 63.4% in Nepal (95% CI = 61.3–65.5). Readiness-adjusted ANC8+ coverage was low across all countries, below 10%, reflecting low coverage of ANC8+ (**Table 1**, **Figure 1**).

### Effective coverage cascade for ANC services

**Figure 2:**
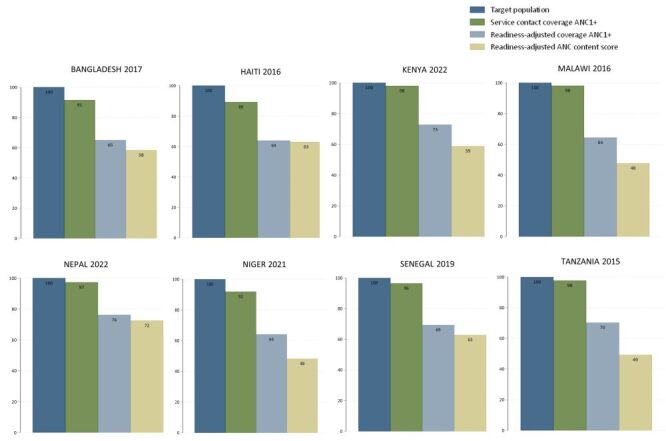
Antenatal care effective coverage cascade, by country. ANC – antenatal care, ANC1+ – at least one antenatal care contact.

The effective coverage cascade for ANC uncovered little lack of access or awareness, with high ANC1+ coverage in all countries except in Haiti where the difference between the population in need (100%) and ANC1+ coverage (89.2%) was of 10.8 percentage points (pp). However, we found large readiness gaps between service contact coverage of ANC1+ and readiness-adjusted ANC1+ coverage in all settings, ranging from 21.1 pp in Nepal, to 33.7 pp in Malawi (**Figure 2**). This indicates that while ANC1+ coverage was near universal in the countries studied, after adjusting for the facilities’ readiness to offer high-quality ANC, the effective coverage was much lower. The cascade further depicts substantial missed opportunities in terms of the content of care received. The readiness-adjusted content coverage of ANC was even lower, ranging from 47.8% in Malawi to 72.5% in Nepal, suggesting missed opportunities as high as 21.1 pp in Tanzania (**Table 1****,** **Figure 2**).

### Socioeconomic inequalities in ANC1+ coverage and readiness-adjusted coverage

Across all countries, the median absolute difference in ANC1+ coverage between urban and rural women was of 2.3 pp, and that of readiness-adjusted ANC1+ was of 6.4 pp. By wealth quintile, the difference in ANC1+ and readiness-adjusted ANC1+ between the richest and poorest women was of 8.5 pp and 14.6 pp respectively.

**Figure 3:**
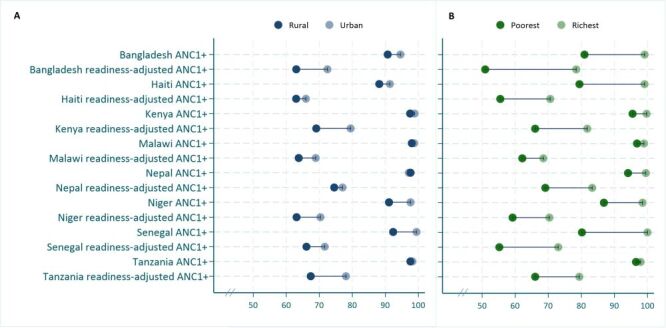
Inequalities in ANC1+ and readiness-adjusted ANC1+ coverage (%) by women’s place of residence (**Panel A**) and wealth quintile (**Panel B**). ANC – antenatal care, ANC1+ – at least one antenatal care contact.

In most countries, while the difference in ANC1+ coverage between urban and rural residents was small, that of readiness-adjusted ANC1+ coverage was much larger. For instance, in Tanzania the ANC1+ inequality (the absolute difference in coverage) between urban and rural residents was of 0.6 pp (ANC1+ coverage of 98.2% among urban and 97.6% among rural residents), whereas that of readiness-adjusted ANC1+ was of 10.6 pp (78.1% among urban and 67.4% among rural) (**Figure 3****,** Panel A). This suggests that while nearly all rural women had an ANC service contact, the readiness of facilities accessed was worse in rural compared to urban areas. Similarly, there were large inequalities between the richest and poorest wealth quintiles both in terms of service contact for ANC and even more so in terms of readiness-adjusted ANC. Various patterns emerge across countries: the first is that of small wealth inequalities in ANC1+ but large wealth inequalities in readiness-adjusted ANC1+ as observed in Kenya, Nepal and Tanzania. The second pattern is that of large wealth inequalities in both ANC1+ and readiness-adjusted ANC1+ in Bangladesh, Haiti, Niger, and Senegal. For instance, in Bangladesh, the ANC1+ coverage gap between richest and poorest households was of 18.2 pp, and that of readiness-adjusted ANC1+ was of 27.5 pp. The third pattern specific to Malawi is that of small wealth inequalities in both ANC1+(2.1 pp) and readiness-adjusted ANC1+ (6.4 pp) (**Figure 3****,** Panel B).

## DISCUSSION

In this study, we ecologically linked 29 925 women’s report of ANC from household surveys with 8621 facilities using health facility assessment data to generate effective coverage cascades for ANC in eight LMICs. Our analysis points to suboptimal readiness-adjusted ANC coverage, despite near universal service contact with health facilities. Readiness-adjusted ANC1+ coverage ranged from 64.0% in Haiti to 76.2% in Nepal, uncovering readiness gaps as large as 33.7 pp in Malawi, as well as substantial missed opportunities. In line with our analysis, other authors have also reported poor quality-adjusted coverage measures of health services along the continuum of care: for instance, Hazel et al. used household survey data to measure quality family planning use in 33 LMICs and found a median quality gap of 27 pp [13]. Similarly, Niehaus et al. found that the coverage of institutional deliveries in Malawi, Mozambique and Tanzania decreased significantly after adjusting for the facilities’ readiness to manage care for the small and sick newborns [14].

Moreover, we found that while ANC 1+ was nearly universal, women face challenges to achieve ANC4+ and the WHO recommended ANC8+ for a positive pregnancy experience [4]. The WHO recommended ANC8+ was introduced in 2016, hence some countries might not have implemented it at the time of the survey, possibly explaining the low coverage in our analysis. Nevertheless, the gap between ANC1+, ANC4+ and ANC8+ has been reported previously, including in previous evidence from 54 LMICs suggesting that initiating ANC within the first trimester significantly predicted a higher number ANC contacts received during pregnancy [15]. Services must continue to maximise contact with health facilities in order to provide all the care necessary to improve effective coverage of ANC.

In terms of facility readiness for ANC, our analysis unveiled key readiness limitations in areas of diagnostic testing as well as poor availability of trained human resources in ANC. For instance, the diagnostics readiness score in Bangladesh was 17.5%, and less than 20% of facilities had the available materials for haemoglobin and syphilis testing. Furthermore, the proportion of staff providing ANC that received training in the previous two years was below 40% across all countries, reflecting poor health worker capacity (**Table 2**; Figure S3 in the **Online Supplementary Document**). Another study evaluating facility readiness for ANC in Haiti, Malawi and Tanzania estimated a mean readiness score ranging from 52.3 to 75.9%, and also pointed to important gaps in human resources and poor availability of trained and motivated staff, further reflecting the shortage of skilled staff in these settings [12]. In Malawi, an analysis of ANC readiness found that while the majority of facilities reported providing routine breastfeeding counselling as part of ANC, only 29% of ANC providers had received recent training related to breastfeeding [16]. The majority of these studies reported improved readiness for maternal and newborn health services in higher-level facilities compared to lower-level facilities [17–19]. Yet, the vast majority of women receive such services in lower-level facilities, as evidenced in our study. Improving facility readiness for ANC is critical, especially in these facilities serving the majority of women in LMICs settings. Moreover, while health facility assessments can inform on whether providers received recent training in a given service area, they do not capture the content of training, nor do they assess providers’ capacity to provide care. Additional efforts are needed to fill this important measurement gap.

Furthermore, our analysis indicated large inequalities in ANC1+ coverage and readiness-adjusted coverage by women’s residence and wealth status. In Bangladesh for instance, the readiness-adjusted ANC1+ absolute inequality between richest and poorest households was of 27.5 pp, indicating that the poorest populations not only lag in terms of accessing services, but when they do access care, it is of much poorer quality and completeness than their richer counterparts, thus pointing to a double disadvantage, or double burden of poor access and poor quality of care. Additional analyses using the Bangladesh Service Provision Assessment (SPA) 2017 confirmed large differences in facility readiness between public and private facilities: the estimated ANC readiness in public facilities was of 56.5% compared to 81% in the private-for-profit sector. Furthermore, Bangladesh’s DHS 2017 data indicated that 39% of the poorest women reported attending ANC in a private hospital, compared to 77.2% of their richest counterparts. These results are consistent with other findings of ‘poor quality for poor women’: in their analysis of facility readiness as well as observations of antenatal and delivery care, Sharma et al. found that the care available to women living in impoverished areas of Kenya was of significantly poorer quality than that available in higher income areas [20]. In our analysis in Kenya, Nepal and Tanzania, we found large inequality in readiness-adjusted ANC1+ coverage between richest and poorest households, while that of ANC1+ coverage was smaller. This confirms that while the poorest women receive ANC services, the facilities they attend have lower readiness for quality care compared to those attended by their wealthier counterparts. Similar results have been reported in other settings: in a study in Mexico, poverty predicted poor ANC quality among rural residents, and in India, providers attended by poorer households were found to have lower competency than those attending richer households [21,22]. Additional research exploring the intersectionality of wealth, residence and subnational region would be beneficial to further complement our findings, and identify the marginalised groups left behind for more targeted action.

Our analysis has strengths and limitations. The use of a linking methodology between household survey and health facility data allows to measure readiness gaps and is a stronger methodology than the use of household survey data alone to approximate readiness or quality of care. Some limitations worth mentioning include possible recall bias as coverage measures rely on women’s self-report; we minimised recall bias by restricting the analysis to women who gave birth two years preceding the survey. Health facility data used rely on facility inventory which reflects availability of items on the day of the survey, and might not be representative of longer periods of time. Similarly, the same items were used to compute ANC readiness scores regardless of facility type, therefore lower-level facilities that do not offer diagnostic testing onsite inadvertently scored lower on such items. The readiness index reflects all items needed for a facility to offer complete and high-quality ANC, with the assumption that service readiness can inform quality of care, and subsequently health gains. This assumption has not been tested here, and while some studies have raised concerns of the lack of an association with quality of care, one study found a small and significant association between facility readiness and provision of ANC services [23]. Additionally, our assumption that facility readiness remains stable over a 2–3-year-period might not always hold true; however, recent evidence suggests that facility readiness for maternal and newborn health services does not fluctuate significantly over that time period [10,11]. Lastly, the ANC content score included six interventions that ought to be received during ANC, based on data availability, and was not an exhaustive list of all ANC interventions.

## CONCLUSIONS

In conclusion, our effective coverage cascade for ANC services sheds light on important obstacles in achieving quality ANC in eight LMICs: while service contact with health facilities appears to be high, readiness-adjusted ANC measures are consistently lower in all countries, pointing to substantial readiness gaps and missed opportunities, driven by suboptimal facility readiness and incomplete content of care. These gaps are disproportionately larger among poorest households and women living in rural areas, complementing previous reports of poor quality among poor women. Exploring the intersectionality of wealth, residence and geographic regions would be beneficial to identify marginalised groups left behind. Further research is needed to contextualise our findings, understand the reasons for the readiness gaps identified in our analysis and the effect on provision of care, through qualitative inquiries with health providers and policy makers, as well as using observational data on care processes. Moreover, there is a need to include the measurement of health providers’ knowledge and capacity in health facility assessments. Strengthening facilities’ diagnostic capacity and availability of skilled and trained human resources will be critical to provide quality ANC and ensure health gains.

## Additional material


Online Supplementary Document
