# Supplementary material for: Building an effective coverage cascade for antenatal care: linking of household survey and health facility assessment data in eight low- and middle-income countries
Source: J Glob Health. 2025 Feb 14;15:04048. doi: 10.7189/jogh.15.04048 (PMC11826959; doi:10.7189/jogh.15.04048)

Building an Effective Coverage Cascade for Antenatal Care:

Linking of Household Survey and Health Facility Assessment Data in Eight Low- and Middle-income Countries.

ONLINE SUPPLEMENTARY DOCUMENT

Supplementary Table S1. Definition of items for ANC readiness index, by domain.

Domain	Item	Definition
Equipment	Blood pressure apparatus	Observed availability of functioning digital or manual blood pressure apparatus (if manual, stethoscope was also available) in general outpatient or ANC area.
	Stethoscope	Observed availability of functioning stethoscope in general outpatient or ANC area.
	Latex gloves	Observed availability of latex gloves in general outpatient or ANC area.
	Examination bed*	Observed availability of examination bed in general outpatient or ANC area.
	Single use syringe	Observed availability of single use or auto-disable syringe in general outpatient or ANC area.
	Soap and water or alcohol-based rub	Observed availability of soap and running water or alcohol-based hand rub in general outpatient or ANC area.
	Environmental disinfectant	Observed availability of an environmental disinfectant in general outpatient or ANC area.
Diagnostics	Hemoglobin testing	Observed availability and functionality of any valid hemoglobin tests: rapid test, hematology analyzer, colorimeter or hemoglobinometer and drabkin's solution with pipette, litmus paper, hemocue and microcuvette; anywhere in facility.
Diagnostics	Urine dipstick protein	Observed availability of valid urine protein dipstick; anywhere in facility.
Diagnostics	Urine dipstick glucose	Observed availability of valid urine glucose dipstick; anywhere in facility.
Diagnostics	Syphilis testing	Observed availability of any valid syphilis tests: rapid test (RDT), PCR, RPR/VDRL and rotator; anywhere in facility.
Diagnostics	HIV testing**	Observed availability and functionality of any valid HIV test: rapid test (RDT) or Elisa equipment; anywhere in facility.

Medicines & commodities[+]	Iron tablets	Observed availability of valid iron tablets (or combined with other minerals); anywhere in facility.
	Folic acid tablets	Observed availability of valid folic acid tablets (or combined with other minerals); anywhere in facility.
	Tetanus toxoid vaccine	Observed availability of valid tetanus toxoid vaccine; anywhere in facility.
Basic amenities	Improved water source	Observed availability of water from the following sources and on-site of within 500m from facility: piped, public tap, protected well, tube well/borehole, protected spring, rain, bottled; anywhere in facility.
	Room with auditory and visual privacy	Observed availability of private room with auditory and visual privacy; outpatient or ANC area.
	Improved sanitation facilities	Observed availability of improved sanitation facilities: flush to piped, flush to tank, flush to latrine, flush to somewhere else, ventilated improved pit latrine, pit latrine with slab, or composting toilet; anywhere in facility
Human resources***	Trained staff in ANC	Proportion of staff providing ANC services trained in ANC in the two years preceding the survey; ANC area.

Note: Where relevant, if an item was not collected in the outpatient/ANC areas, we included other areas (delivery, infection diseases, etc.)
**Examination bed not collected in Niger and Kenya*
*** HIV diagnostics not included in Bangladesh*
+ tetanus toxoid vaccine not collected in Kenya
**** trained staff in ANC not collected in Kenya*

Supplementary Figure S1. Place of ANC among all women with a birth 2 years preceding survey.

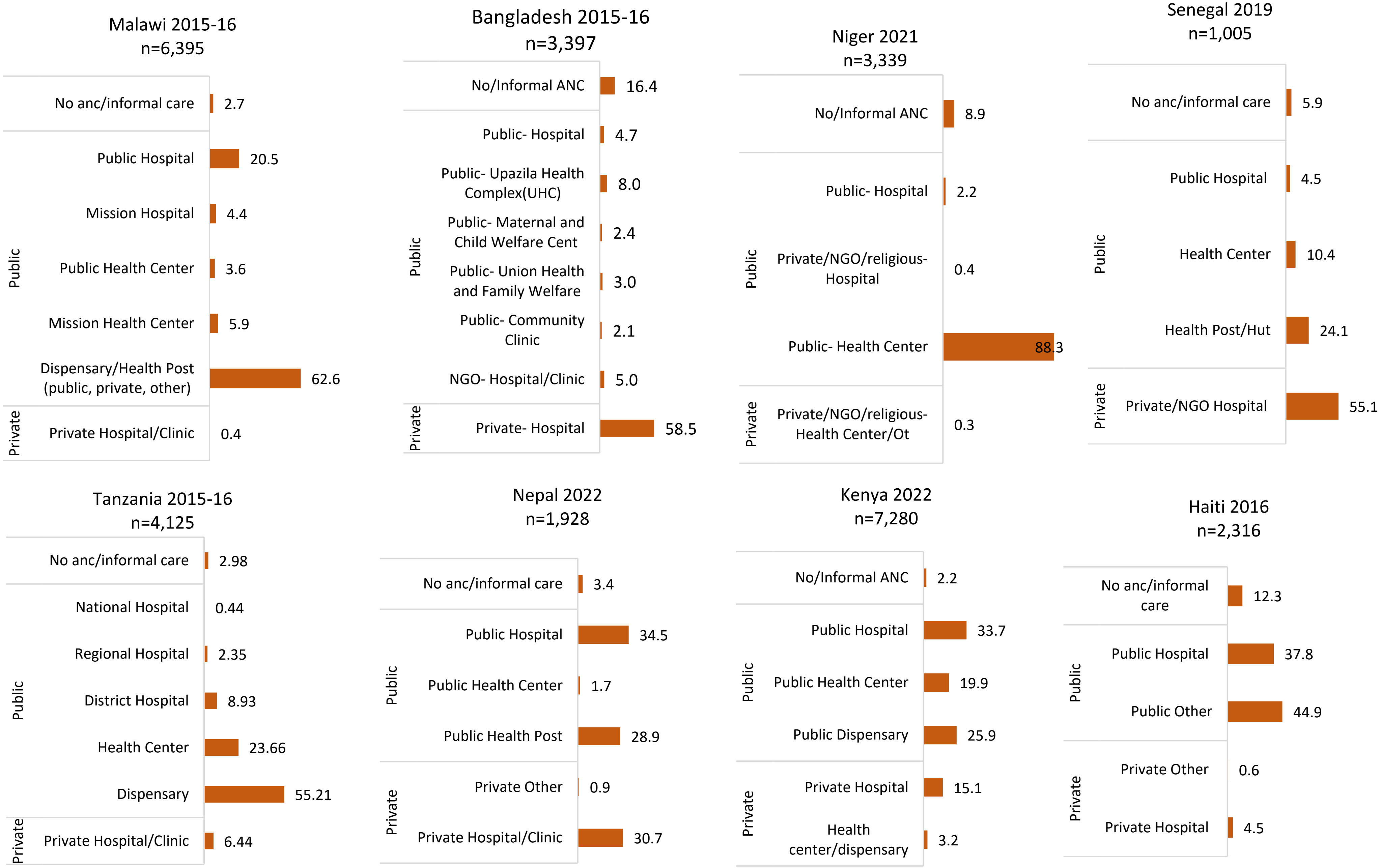

Supplementary Figure S2. ANC facility readiness score by country and facility type/managing authority (N= number of facilities).

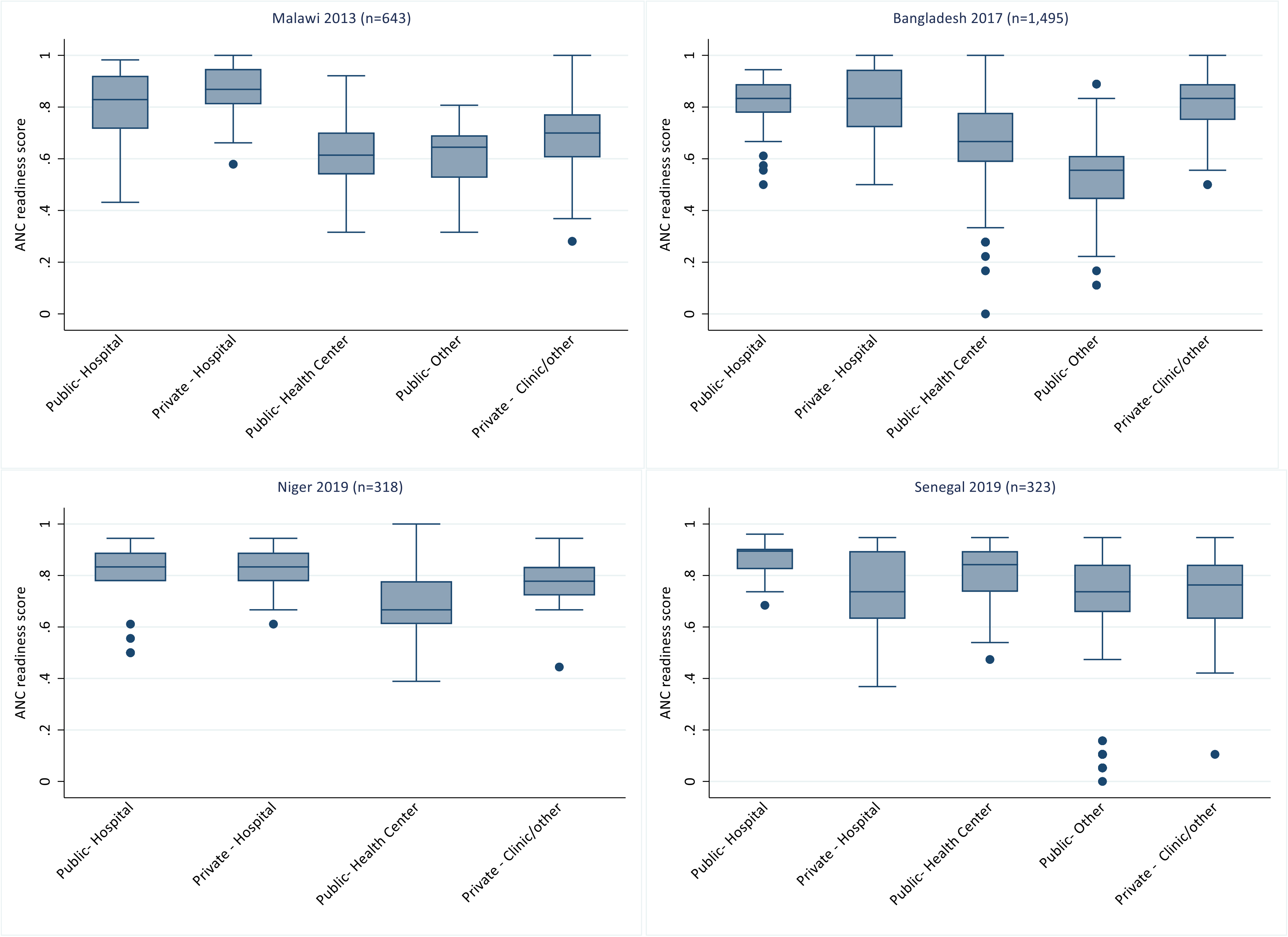

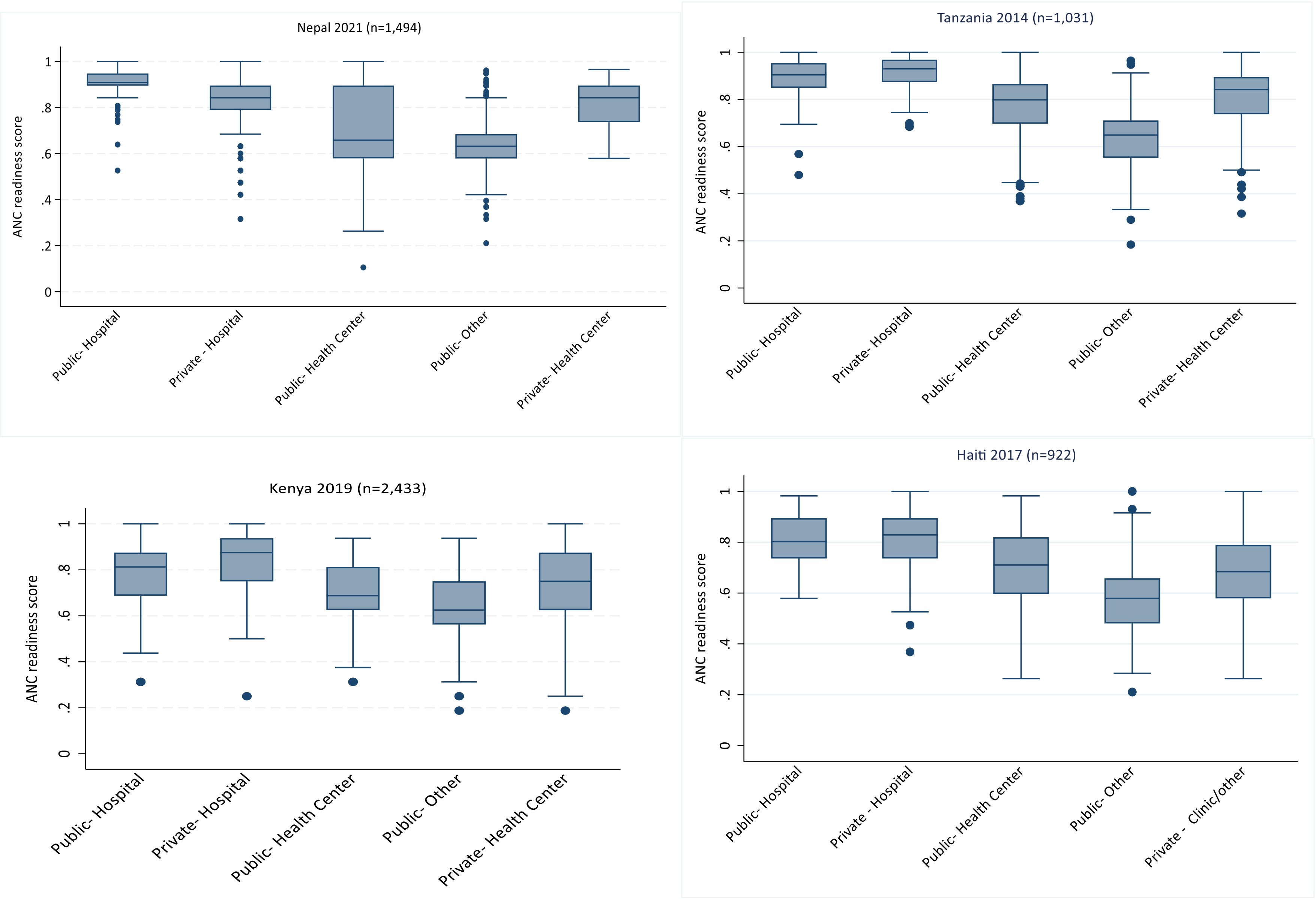

Nepal 2021 (n=1,494)
ANC readiness score
1
.8
.6
.4
.2
0
Public- Hospital
Private - Hospital
Public- Health Center
Public- Other
Private- Health Center
Tanzania 2014 (n=1,031)
ANC readiness score
1
.8
.6
.4
.2
0
Public- Hospital
Private- Hospital
Public- Health Center
Public- Other
Private- Health Center
Kenya 2019 (n=2,433)
ANC readiness score
1
.8
.6
.4
.2
0
Public- Hospital
Private- Hospital
Public- Health Center
Public- Other
Private- Health Center
Haiti 2017 (n=922)
ANC readiness score
1
.8
.6
.4
.2
0
Public- Hospital
Private - Hospital
Public- Health Center
Public- Other
Private - Clinic/other

Supplementary figure S3. Availability of ANC items by domain and country.

Malawi

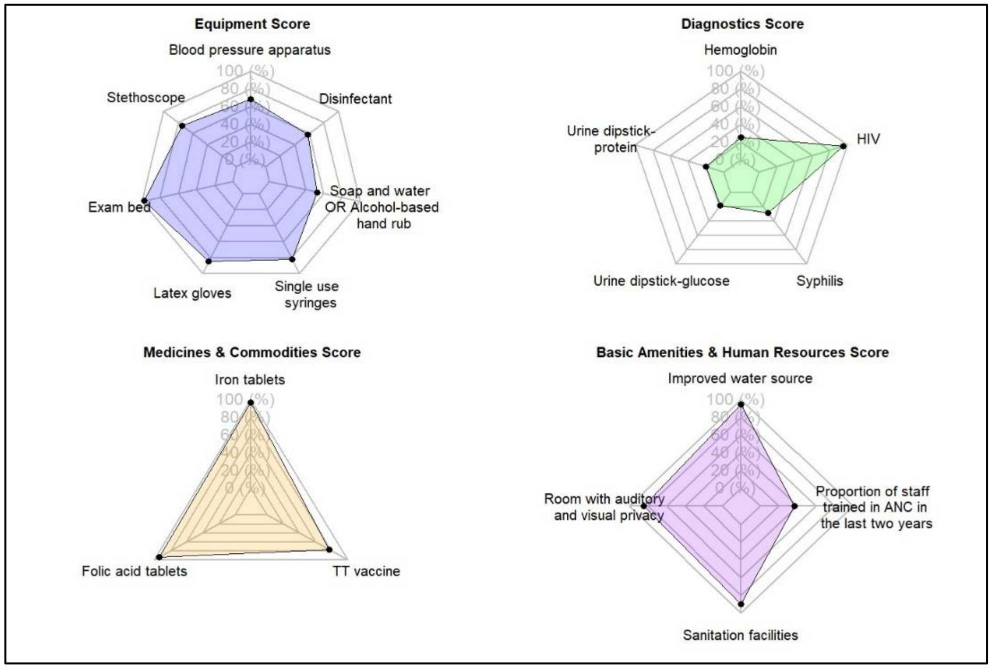

Bangladesh

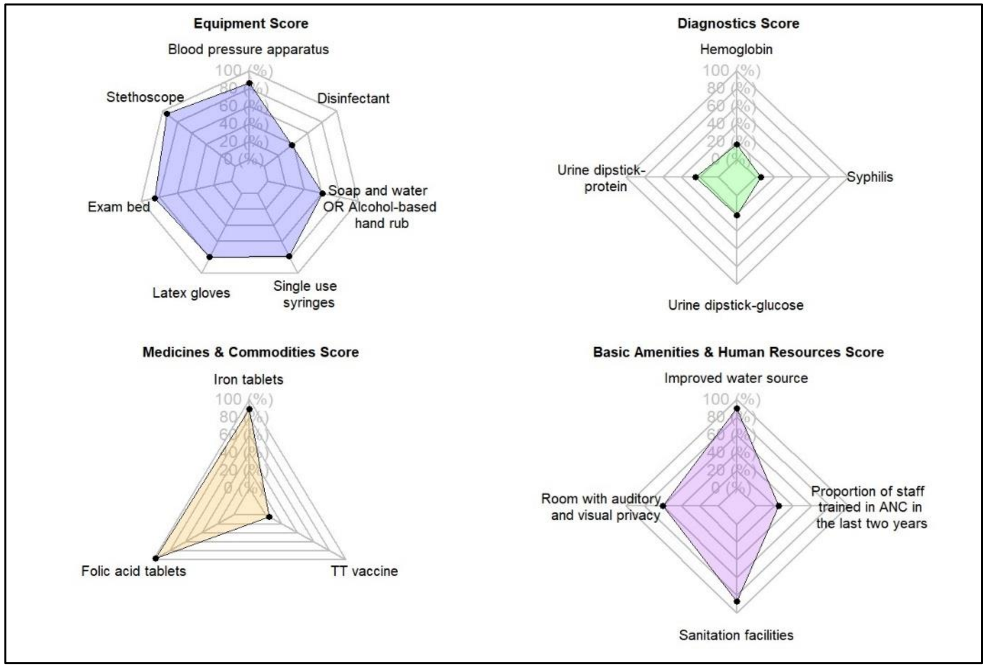

Niger

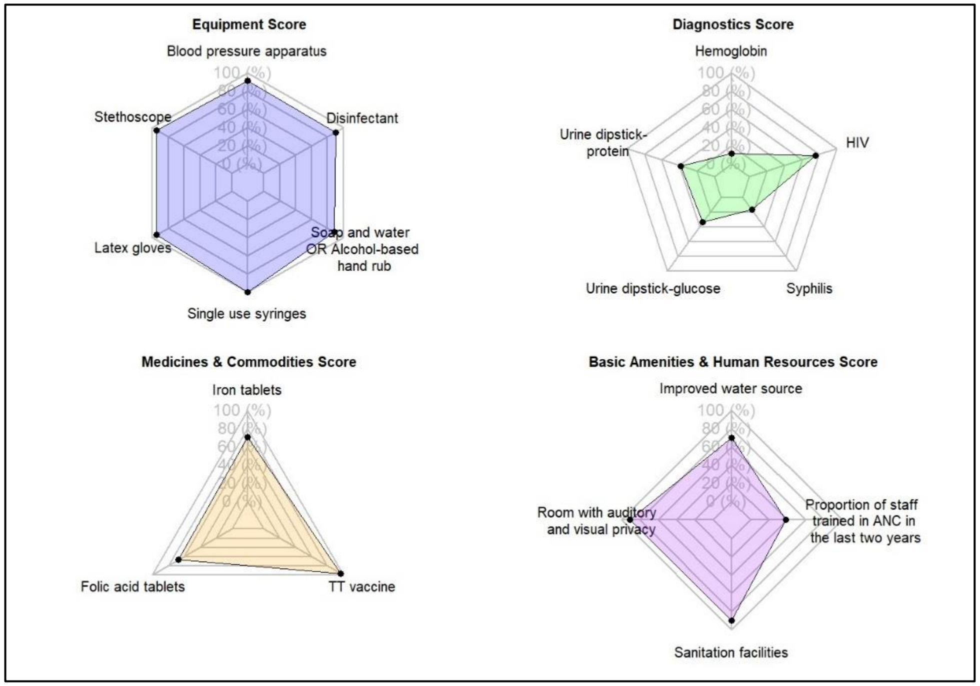

Senegal

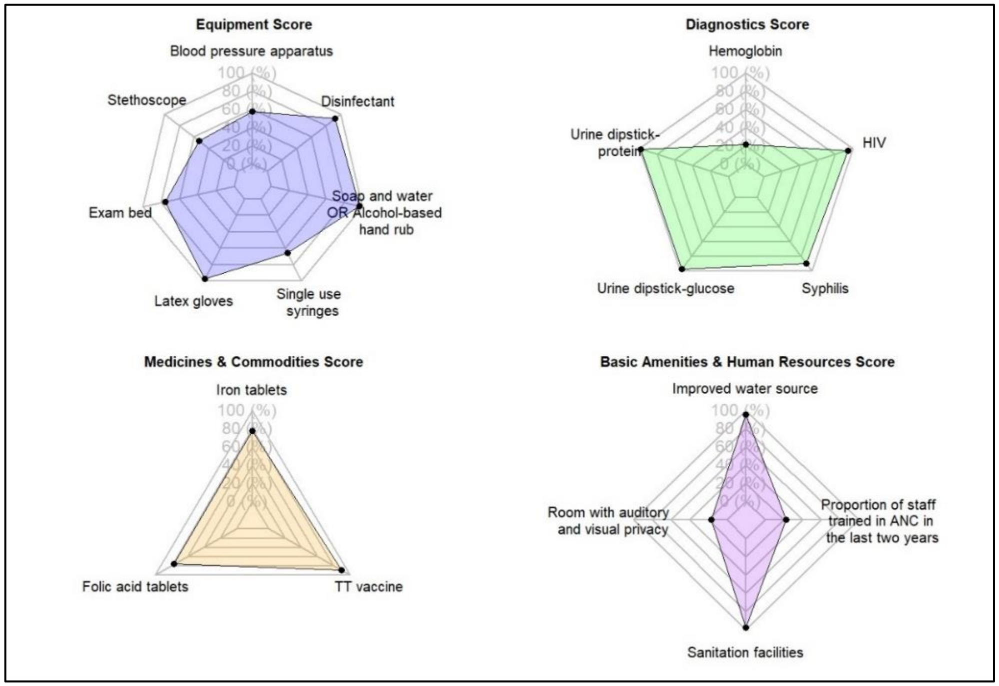

Nepal

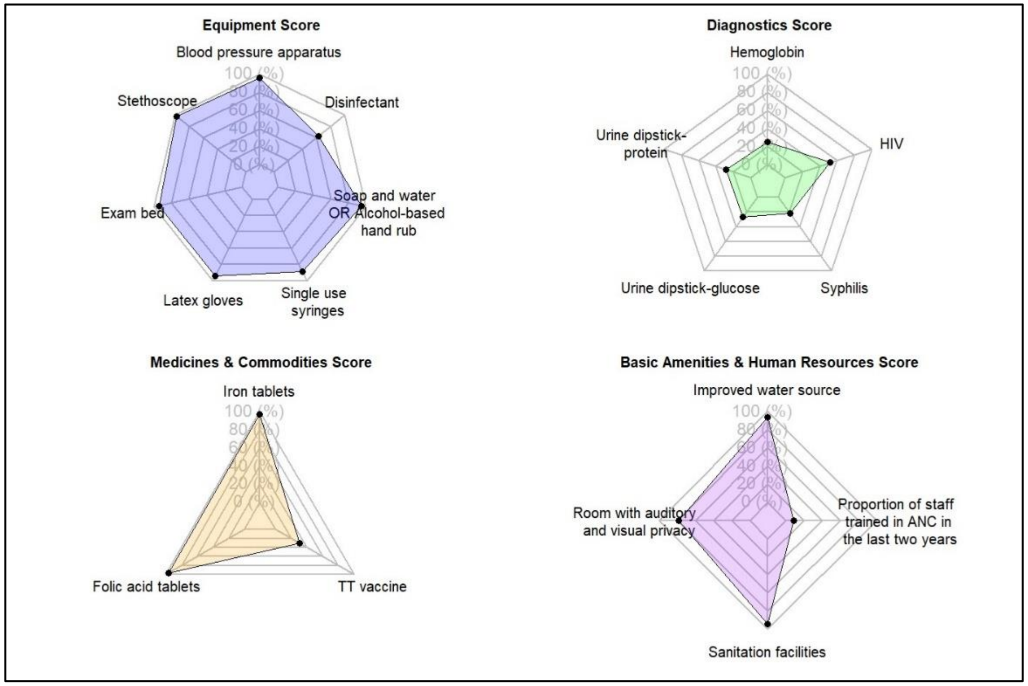

Tanzania

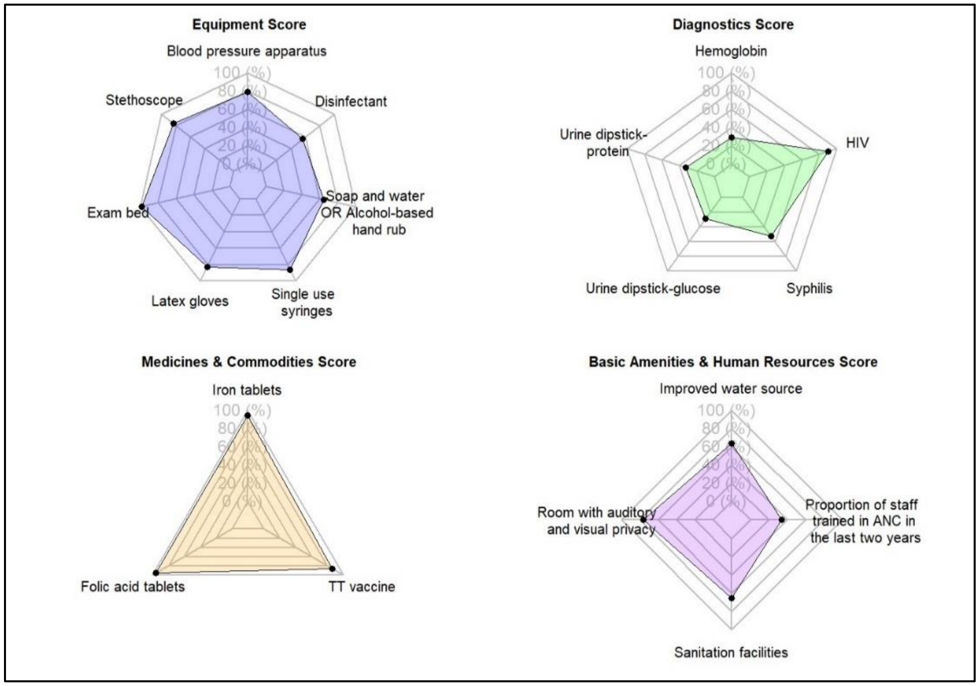

Kenya

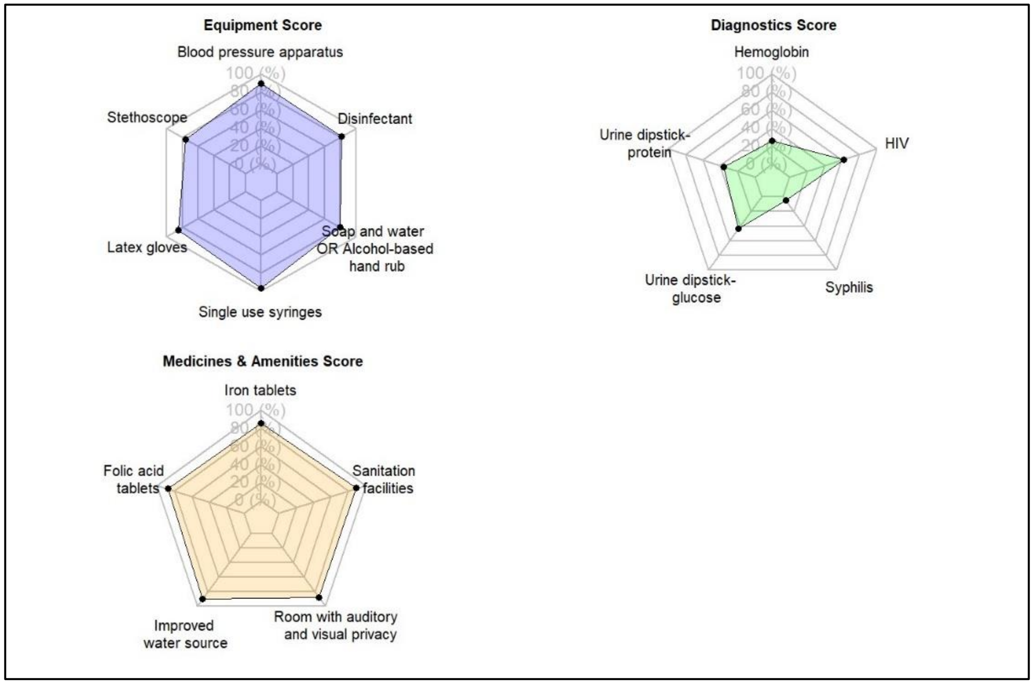

Haiti

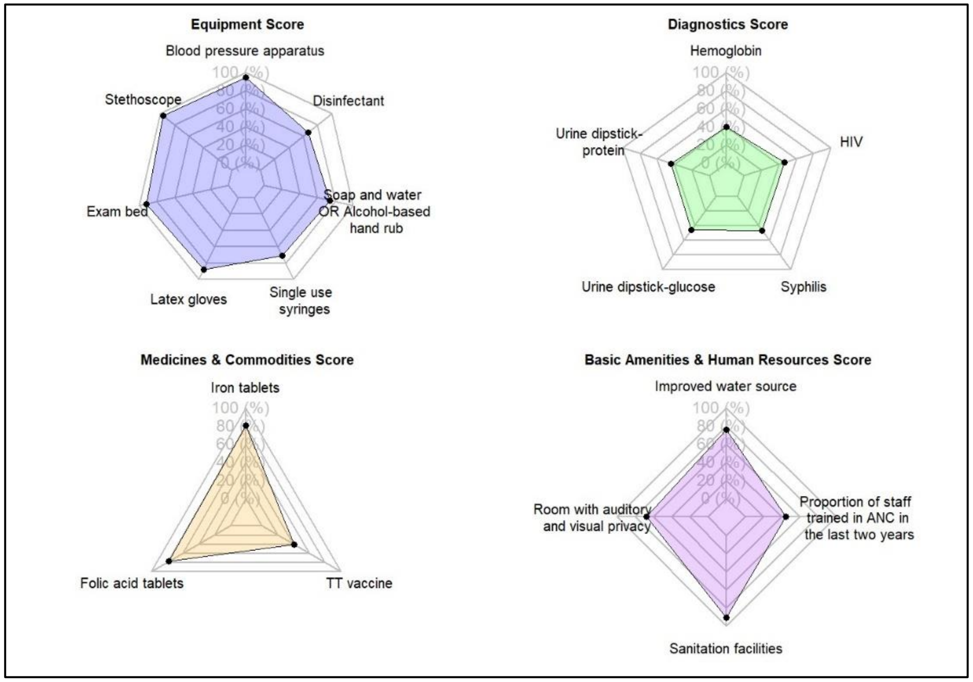

Supplement: Online Supplementary Document [file jogh-15-04048-s001.pdf]
